# Dichlorido{2-[(2-isopropyl­ammonio­ethyl)imino­meth­yl]-5-methoxy­phenolato}zinc(II)

**DOI:** 10.1107/S160053681001127X

**Published:** 2010-03-31

**Authors:** Zhen-Quan Han, Yuan Wang, Shuang Han

**Affiliations:** a Applied Technical College, Qiqihar University, Qiqihar 161006, People’s Republic of China; b Qiqihar Environmental Monitoring Central Station, Qiqihar 161005, People’s Republic of China; c College of Chemistry and Chemical Engineering, Qiqihar University, Qiqihar 161006, People’s Republic of China

## Abstract

The Zn^II^ atom in the title compound, [ZnCl_2_(C_13_H_20_N_2_O_2_)], is four-coordinated by the imine N and phenolate O atoms of the zwitterionic Schiff base ligand, and by two choride ions in a distorted tetra­hedral coordination. In the crystal structure, mol­ecules are linked through inter­molecular N—H⋯O and N—H⋯Cl hydrogen bonds along [010].

## Related literature

For a nickel(II) complex with the 3-ethoxy­salicylaldehyde ligand, see: Han (2008 ▶). For similar zinc(II) complexes with Schiff bases, see: Ali *et al.* (2008 ▶); Wang (2007 ▶); Zhang *et al.* (2008 ▶); Zhu *et al.* (2009 ▶).
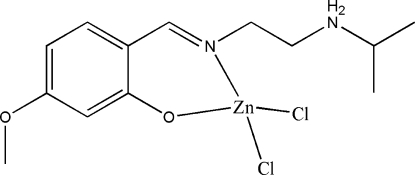


## Experimental

### 

#### Crystal data

[ZnCl_2_(C_13_H_20_N_2_O_2_)]
*M* *_r_* = 372.58
Monoclinic, 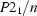

*a* = 6.2915 (9) Å
*b* = 11.8990 (18) Å
*c* = 22.115 (4) Å
β = 96.518 (4)°
*V* = 1644.9 (4) Å^3^
*Z* = 4
Mo *K*α radiation
μ = 1.82 mm^−1^
*T* = 298 K
0.18 × 0.18 × 0.18 mm

#### Data collection

Bruker SMART CCD area-detector diffractometer
Absorption correction: multi-scan (*SADABS*; Sheldrick, 1996 ▶) *T*_min_ = 0.735, *T*_max_ = 0.735
9437 measured reflections
3557 independent reflections
2515 reflections with *I* > 2σ(*I*)
*R*_int_ = 0.042

#### Refinement

*R*[*F* ^2^ > 2σ(*F* ^2^)] = 0.038
*wR*(*F* ^2^) = 0.086
*S* = 1.01
3557 reflections
184 parameters
H-atom parameters constrained
Δρ_max_ = 0.46 e Å^−3^
Δρ_min_ = −0.31 e Å^−3^

### 

Data collection: *SMART* (Bruker, 1998 ▶); cell refinement: *SAINT* (Bruker, 1998 ▶); data reduction: *SAINT*; program(s) used to solve structure: *SHELXS97* (Sheldrick, 2008 ▶); program(s) used to refine structure: *SHELXL97* (Sheldrick, 2008 ▶); molecular graphics: *SHELXTL* (Sheldrick, 2008 ▶); software used to prepare material for publication: *SHELXTL*.

## Supplementary Material

Crystal structure: contains datablocks global, I. DOI: 10.1107/S160053681001127X/bx2273sup1.cif

Structure factors: contains datablocks I. DOI: 10.1107/S160053681001127X/bx2273Isup2.hkl

Additional supplementary materials:  crystallographic information; 3D view; checkCIF report

## Figures and Tables

**Table:** 

Zn1—O1	1.9425 (19)
Zn1—N1	1.997 (2)
Zn1—Cl2	2.2290 (9)
Zn1—Cl1	2.2554 (10)

**Table:** 

O1—Zn1—N1	97.16 (9)
O1—Zn1—Cl2	107.02 (6)
N1—Zn1—Cl2	113.82 (7)
O1—Zn1—Cl1	111.15 (7)
N1—Zn1—Cl1	110.44 (7)
Cl2—Zn1—Cl1	115.64 (3)

**Table 2:** Hydrogen-bond geometry (Å, °)

*D*—H⋯*A*	*D*—H	H⋯*A*	*D*⋯*A*	*D*—H⋯*A*
N2—H2*A*⋯Cl2^i^	0.90	2.62	3.357 (2)	140
N2—H2*B*⋯O1^i^	0.90	1.91	2.782 (3)	162
N2—H2*A*⋯Cl1	0.90	2.97	3.483 (2)	118

Symmetry code: (i) 
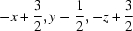
.

**Fig. 1.**
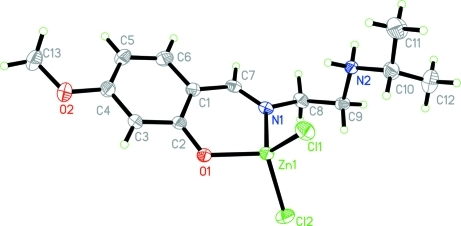
The structure of the title complex, showing 30% probability displacement ellipsoids and the atom-numbering scheme.

**Fig. 2.**
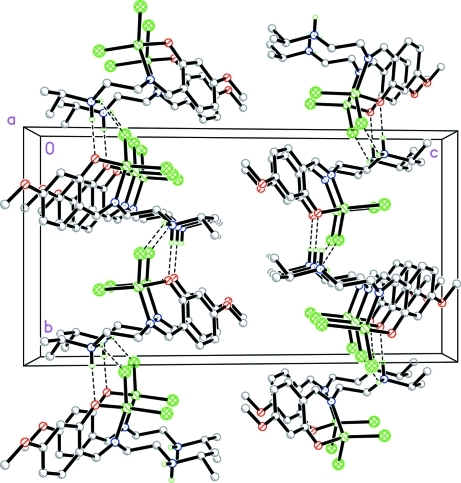
Molecular packing of the title complex.

## supplementary crystallographic information

### Comment

Recently, we have reported a nickel(II) complex with the ligand 3-ethoxysalicylaldehyde (Han, 2008). We report here a new zinc(II) complex derived from the Schiff base ligand 2-[(2-isopropylammonioethylimino)methyl]-5-methoxyphenol, which was formed by the condensation reaction of 4-methoxysalicylaldehyde with *N*-isopropylethane-1,2-diamine in a methanol solution.The Zn^II^ atom is four-coordinated by the imine N and phenolate O atoms of the Schiff base ligand, and by two choride ions in a distorted tetrahedral coordination. In the crystal structure, molecules are linked through intermolecular N—H··· O and N—H ···O and hydrogen bonds along [010], (Fig. 2). The geometric parameters are comparable to those in similar zinc(II) complexes with Schiff bases (Wang, 2007; Ali *et al.*, 2008; Zhang *et al.*, 2008; Zhu *et al.*, 2009) as representative examples.

### Experimental

All the chemicals were of AR grade. 4-Methoxysalicylaldehyde (30.4 mg, 0.2 mmol) and *N*-isopropylethane-1,2-diamine (20.4 mg, 0.2 mmol) were stirred in a methanol solution for 2 h at reflux. Then the zinc(II) chloride (27.5 mg, 0.2 mmol) was added to the mixture. The mixture was further stirred at reflux for 1 h. The mixture was cooled to room temperature and filtered. After keeping the filtrate in air for a week, yielding colorless block crystals suitable for X-ray analysis.

### Refinement

H atoms were placed in idealized positions and constrained to ride on their parent atoms with C–H distances in the range 0.93-0.97 Å, N–H distances of 0.90 Å, and with U_iso_(H) set at 1.2U_eq_(C,*N*) and 1.5U_eq_(methyl C).

### Crystal data

**Table:**

[ZnCl_2_(C_13_H_20_N_2_O_2_)]	*F*(000) = 768
*M**_r_* = 372.58	*D*_x_ = 1.504 Mg m^−^^3^
Monoclinic, *P*2_1_/*n*	Mo *K*α radiation, λ = 0.71073 Å
*a* = 6.2915 (9) Å	Cell parameters from 1935 reflections
*b* = 11.8990 (18) Å	θ = 2.5–24.5°
*c* = 22.115 (4) Å	µ = 1.82 mm^−^^1^
β = 96.518 (4)°	*T* = 298 K
*V* = 1644.9 (4) Å^3^	Block, colourless
*Z* = 4	0.18 × 0.18 × 0.18 mm

### Data collection

**Table:**

Bruker SMART CCD area-detector diffractometer	3557 independent reflections
Radiation source: fine-focus sealed tube	2515 reflections with *I* > 2σ(*I*)
graphite	*R*_int_ = 0.042
ω scans	θ_max_ = 27.0°, θ_min_ = 1.9°
Absorption correction: multi-scan (*SADABS*; Sheldrick, 1996)	*h* = −7→8
*T*_min_ = 0.735, *T*_max_ = 0.735	*k* = −13→15
9437 measured reflections	*l* = −27→28

### Refinement

**Table:**

Refinement on *F*^2^	Primary atom site location: structure-invariant direct methods
Least-squares matrix: full	Secondary atom site location: difference Fourier map
*R*[*F*^2^ > 2σ(*F*^2^)] = 0.038	Hydrogen site location: inferred from neighbouring sites
*wR*(*F*^2^) = 0.086	H-atom parameters constrained
*S* = 1.01	*w* = 1/[σ^2^(*F*_o_^2^) + (0.0339*P*)^2^ + 0.3384*P*] where *P* = (*F*_o_^2^ + 2*F*_c_^2^)/3
3557 reflections	(Δ/σ)_max_ = 0.001
184 parameters	Δρ_max_ = 0.46 e Å^−^^3^
0 restraints	Δρ_min_ = −0.31 e Å^−^^3^

### Special details

**Table:**

Geometry. All esds (except the esd in the dihedral angle between two l.s. planes) are estimated using the full covariance matrix. The cell esds are taken into account individually in the estimation of esds in distances, angles and torsion angles; correlations between esds in cell parameters are only used when they are defined by crystal symmetry. An approximate (isotropic) treatment of cell esds is used for estimating esds involving l.s. planes.
Refinement. Refinement of *F*^2^ against ALL reflections. The weighted *R*-factor wR and goodness of fit *S* are based on *F*^2^, conventional *R*-factors *R* are based on *F*, with *F* set to zero for negative *F*^2^. The threshold expression of *F*^2^ > σ(*F*^2^) is used only for calculating *R*-factors(gt) etc. and is not relevant to the choice of reflections for refinement. *R*-factors based on *F*^2^ are statistically about twice as large as those based on *F*, and *R*- factors based on ALL data will be even larger.

### Fractional atomic coordinates and isotropic or equivalent isotropic displacement parameters (Å^2^)

**Table:**

	*x*	*y*	*z*	*U*_iso_*/*U*_eq_	
Zn1	0.75702 (6)	0.83340 (3)	0.763592 (16)	0.03646 (12)	
Cl1	0.56343 (14)	0.81826 (7)	0.67166 (4)	0.0519 (2)	
Cl2	1.00002 (12)	0.97048 (6)	0.77248 (4)	0.0497 (2)	
N1	0.8725 (4)	0.68364 (18)	0.79167 (11)	0.0336 (6)	
N2	0.9034 (4)	0.58914 (18)	0.66336 (10)	0.0329 (6)	
H2A	0.7788	0.5959	0.6794	0.039*	
H2B	0.9412	0.5162	0.6658	0.039*	
O1	0.5732 (3)	0.85623 (15)	0.82740 (9)	0.0399 (5)	
O2	0.0900 (4)	0.7411 (2)	0.95938 (11)	0.0652 (7)	
C1	0.6016 (5)	0.6635 (2)	0.86232 (13)	0.0360 (7)	
C2	0.5081 (5)	0.7727 (2)	0.85973 (13)	0.0336 (7)	
C3	0.3364 (5)	0.7916 (2)	0.89406 (14)	0.0397 (7)	
H3	0.2735	0.8625	0.8931	0.048*	
C4	0.2582 (5)	0.7086 (3)	0.92900 (14)	0.0449 (8)	
C5	0.3475 (6)	0.6021 (3)	0.93171 (16)	0.0524 (9)	
H5	0.2956	0.5460	0.9554	0.063*	
C6	0.5158 (5)	0.5815 (3)	0.89822 (15)	0.0483 (8)	
H6	0.5752	0.5099	0.8995	0.058*	
C7	0.7806 (5)	0.6282 (2)	0.83109 (13)	0.0367 (7)	
H7	0.8353	0.5571	0.8408	0.044*	
C8	1.0626 (5)	0.6333 (3)	0.76875 (14)	0.0417 (8)	
H8A	1.0622	0.5528	0.7758	0.050*	
H8B	1.1907	0.6640	0.7913	0.050*	
C9	1.0685 (5)	0.6552 (2)	0.70159 (14)	0.0374 (7)	
H9A	1.0454	0.7346	0.6935	0.045*	
H9B	1.2089	0.6358	0.6906	0.045*	
C10	0.8660 (6)	0.6211 (3)	0.59723 (15)	0.0513 (9)	
H10	0.8115	0.6983	0.5952	0.062*	
C11	0.6940 (6)	0.5472 (3)	0.56592 (17)	0.0732 (12)	
H11A	0.7428	0.4707	0.5669	0.110*	
H11B	0.6613	0.5709	0.5244	0.110*	
H11C	0.5679	0.5526	0.5864	0.110*	
C12	1.0658 (7)	0.6201 (4)	0.56802 (18)	0.0780 (13)	
H12A	1.1282	0.5465	0.5715	0.117*	
H12B	1.1643	0.6738	0.5878	0.117*	
H12C	1.0346	0.6394	0.5258	0.117*	
C13	0.0014 (7)	0.6613 (3)	0.99717 (18)	0.0725 (12)	
H13A	−0.0495	0.5974	0.9732	0.109*	
H13B	−0.1155	0.6949	1.0150	0.109*	
H13C	0.1092	0.6376	1.0288	0.109*	

### Atomic displacement parameters (Å^2^)

**Table:**

	*U*^11^	*U*^22^	*U*^33^	*U*^12^	*U*^13^	*U*^23^
Zn1	0.0419 (2)	0.02734 (18)	0.0413 (2)	0.00185 (15)	0.00994 (15)	−0.00074 (15)
Cl1	0.0564 (5)	0.0489 (5)	0.0486 (5)	0.0057 (4)	−0.0021 (4)	−0.0049 (4)
Cl2	0.0413 (5)	0.0393 (4)	0.0687 (6)	−0.0057 (4)	0.0072 (4)	0.0064 (4)
N1	0.0342 (14)	0.0289 (13)	0.0369 (14)	0.0010 (10)	0.0005 (11)	−0.0047 (10)
N2	0.0353 (14)	0.0291 (12)	0.0351 (14)	0.0002 (10)	0.0076 (11)	−0.0013 (10)
O1	0.0491 (13)	0.0260 (10)	0.0477 (13)	0.0021 (9)	0.0186 (11)	0.0011 (9)
O2	0.0748 (18)	0.0618 (15)	0.0661 (17)	−0.0106 (13)	0.0389 (15)	−0.0050 (13)
C1	0.0391 (17)	0.0321 (15)	0.0365 (17)	−0.0011 (13)	0.0028 (13)	−0.0009 (13)
C2	0.0367 (17)	0.0316 (16)	0.0320 (17)	−0.0050 (13)	0.0018 (13)	−0.0046 (13)
C3	0.0457 (19)	0.0347 (16)	0.0399 (18)	−0.0030 (14)	0.0095 (15)	−0.0069 (14)
C4	0.047 (2)	0.051 (2)	0.0370 (18)	−0.0116 (16)	0.0082 (15)	−0.0074 (15)
C5	0.061 (2)	0.048 (2)	0.050 (2)	−0.0107 (17)	0.0151 (18)	0.0073 (16)
C6	0.055 (2)	0.0361 (17)	0.054 (2)	−0.0002 (15)	0.0057 (18)	0.0081 (15)
C7	0.0444 (18)	0.0255 (14)	0.0384 (18)	0.0035 (13)	−0.0035 (15)	−0.0021 (13)
C8	0.0338 (17)	0.0439 (18)	0.046 (2)	0.0068 (14)	0.0002 (14)	−0.0072 (14)
C9	0.0297 (15)	0.0320 (16)	0.051 (2)	−0.0015 (13)	0.0076 (14)	−0.0029 (14)
C10	0.069 (2)	0.0459 (19)	0.039 (2)	0.0039 (18)	0.0056 (18)	0.0044 (15)
C11	0.085 (3)	0.084 (3)	0.046 (2)	−0.011 (2)	−0.014 (2)	−0.005 (2)
C12	0.085 (3)	0.095 (3)	0.058 (3)	0.002 (3)	0.025 (2)	0.011 (2)
C13	0.082 (3)	0.080 (3)	0.062 (3)	−0.020 (2)	0.035 (2)	0.000 (2)

### Geometric parameters (Å, °)

**Table:**

Zn1—O1	1.9425 (19)	C5—H5	0.9300
Zn1—N1	1.997 (2)	C6—H6	0.9300
Zn1—Cl2	2.2290 (9)	C7—H7	0.9300
Zn1—Cl1	2.2554 (10)	C8—C9	1.513 (4)
N1—C7	1.282 (3)	C8—H8A	0.9700
N1—C8	1.478 (3)	C8—H8B	0.9700
N2—C9	1.487 (4)	C9—H9A	0.9700
N2—C10	1.504 (4)	C9—H9B	0.9700
N2—H2A	0.9000	C10—C12	1.477 (5)
N2—H2B	0.9000	C10—C11	1.502 (5)
O1—C2	1.317 (3)	C10—H10	0.9800
O2—C4	1.372 (4)	C11—H11A	0.9600
O2—C13	1.420 (4)	C11—H11B	0.9600
C1—C6	1.404 (4)	C11—H11C	0.9600
C1—C2	1.425 (4)	C12—H12A	0.9600
C1—C7	1.448 (4)	C12—H12B	0.9600
C2—C3	1.408 (4)	C12—H12C	0.9600
C3—C4	1.379 (4)	C13—H13A	0.9600
C3—H3	0.9300	C13—H13B	0.9600
C4—C5	1.385 (4)	C13—H13C	0.9600
C5—C6	1.381 (4)		
			
O1—Zn1—N1	97.16 (9)	C1—C7—H7	116.2
O1—Zn1—Cl2	107.02 (6)	N1—C8—C9	112.2 (2)
N1—Zn1—Cl2	113.82 (7)	N1—C8—H8A	109.2
O1—Zn1—Cl1	111.15 (7)	C9—C8—H8A	109.2
N1—Zn1—Cl1	110.44 (7)	N1—C8—H8B	109.2
Cl2—Zn1—Cl1	115.64 (3)	C9—C8—H8B	109.2
C7—N1—C8	118.0 (2)	H8A—C8—H8B	107.9
C7—N1—Zn1	119.60 (19)	N2—C9—C8	111.9 (2)
C8—N1—Zn1	122.37 (19)	N2—C9—H9A	109.2
C9—N2—C10	116.2 (2)	C8—C9—H9A	109.2
C9—N2—H2A	108.2	N2—C9—H9B	109.2
C10—N2—H2A	108.2	C8—C9—H9B	109.2
C9—N2—H2B	108.2	H9A—C9—H9B	107.9
C10—N2—H2B	108.2	C12—C10—C11	113.4 (3)
H2A—N2—H2B	107.4	C12—C10—N2	112.1 (3)
C2—O1—Zn1	122.43 (17)	C11—C10—N2	109.0 (3)
C4—O2—C13	118.4 (3)	C12—C10—H10	107.3
C6—C1—C2	118.2 (3)	C11—C10—H10	107.3
C6—C1—C7	116.0 (3)	N2—C10—H10	107.3
C2—C1—C7	125.8 (3)	C10—C11—H11A	109.5
O1—C2—C3	118.5 (2)	C10—C11—H11B	109.5
O1—C2—C1	123.9 (2)	H11A—C11—H11B	109.5
C3—C2—C1	117.6 (3)	C10—C11—H11C	109.5
C4—C3—C2	122.1 (3)	H11A—C11—H11C	109.5
C4—C3—H3	118.9	H11B—C11—H11C	109.5
C2—C3—H3	118.9	C10—C12—H12A	109.5
O2—C4—C3	114.6 (3)	C10—C12—H12B	109.5
O2—C4—C5	124.6 (3)	H12A—C12—H12B	109.5
C3—C4—C5	120.8 (3)	C10—C12—H12C	109.5
C6—C5—C4	118.1 (3)	H12A—C12—H12C	109.5
C6—C5—H5	121.0	H12B—C12—H12C	109.5
C4—C5—H5	121.0	O2—C13—H13A	109.5
C5—C6—C1	123.2 (3)	O2—C13—H13B	109.5
C5—C6—H6	118.4	H13A—C13—H13B	109.5
C1—C6—H6	118.4	O2—C13—H13C	109.5
N1—C7—C1	127.6 (3)	H13A—C13—H13C	109.5
N1—C7—H7	116.2	H13B—C13—H13C	109.5

### Hydrogen-bond geometry (Å, °)

**Table:**

*D*—H···*A*	*D*—H	H···*A*	*D*···*A*	*D*—H···*A*
N2—H2A···Cl2^i^	0.90	2.62	3.357 (2)	140
N2—H2B···O1^i^	0.90	1.91	2.782 (3)	162
N2—H2A···Cl1	0.90	2.97	3.483 (2)	118

Symmetry codes: (i) −*x*+3/2, *y*−1/2, −*z*+3/2.

## References

[bb1] Ali, H. M., Mohamed Mustafa, M. I., Rizal, M. R. & Ng, S. W. (2008). *Acta Cryst.* E**64**, m718–m719. 10.1107/S1600536808011161 PMC2961161 21202245

[bb2] Bruker (1998). *SMART* and *SAINT* Bruker AXS Inc., Madison, Wisconsin, USA.

[bb3] Han, Z.-Q. (2008). *Acta Cryst.* E**64**, m592. 10.1107/S160053680800809X PMC2960901 21202039

[bb4] Sheldrick, G. M. (1996). *SADABS* University of Göttingen, Germany.

[bb5] Sheldrick, G. M. (2008). *Acta Cryst.* A**64**, 112–122. 10.1107/S0108767307043930 18156677

[bb6] Wang, S.-X. (2007). *Acta Cryst.* E**63**, m706–m707.

[bb7] Zhang, D.-F., Zhou, M.-H. & Yuan, C.-J. (2008). *Acta Cryst.* E**64**, m825–m826. 10.1107/S1600536808014311 PMC2961511 21202508

[bb8] Zhu, X.-W., Yang, X.-Z., Zhang, C.-X., Li, G.-S. & Yin, Z.-G. (2009). *Acta Cryst.* E**65**, m1332–m1333. 10.1107/S1600536809040495 PMC2971035 21578090

